# Cutaneous *Fusarium *infection in a renal transplant recipient: a case report

**DOI:** 10.1186/1752-1947-5-205

**Published:** 2011-05-25

**Authors:** John S Banerji, Chandra Singh J

**Affiliations:** 1 Department of Urology, Unit 1, Christian Medical College, Vellore, India

## Abstract

**Introduction:**

Fungal infections in the immunocompromised host are fairly common. Of the mycoses, *Fusarium *species are an emerging threat. *Fusarium *infections have been reported in solid organ transplants, with three reports of the infection in patients who had received renal transplants. To the best of our knowledge, this is the first case of an isolated cutaneous lesion as the only form of infection.

**Case presentation:**

We report the case of a 45-year-old South Indian man who presented with localized cutaneous *Fusarium *infection following a renal transplant.

**Conclusion:**

In an immunocompromised patient, even an innocuous lesion needs to be addressed with the initiation of prompt treatment.

## Introduction

*Fusarium *species are common soil saprophytes and plant pathogens. Young and Meyers [1] first reported *Fusarium *infection in the late 1970s. Since then, several species have been recognized to be agents of superficial infections (keratitis, cutaneous infections, onychomycosis and infection of wounds or burns) in humans [2]. More recently, deep-seated, disseminated infections have been increasingly described in immunocompromised patients, especially in neutropenic patients [3,4]. The prognosis is very poor, and death occurs in up to 70% of the cases despite antifungal therapy [4]. The *Fusarium *species most frequently involved in human infections are *Fusarium solani*, *F. oxysporum *and *F. moniliforme*.

## Case report

**Figure 1:**
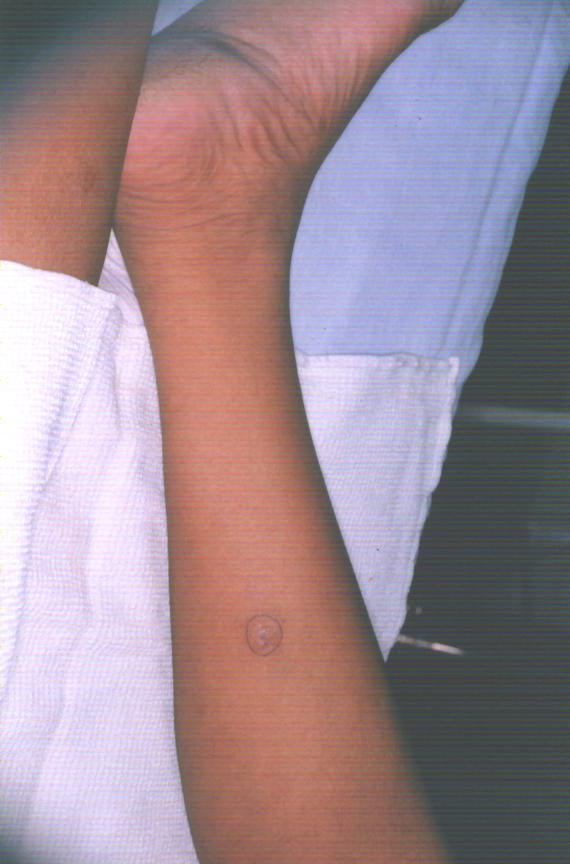
**Nodule on the patient's right calf**.

A 45-year-old South Indian man underwent a renal allograft transplant for end-stage renal disease. He was administered tacrolimus, mycophenolate and prednisolone as immunosuppressive therapy. On follow-up at six months, he complained of a small, painless nodule on his right calf. He had no fever, redness or pruritus. He had no other opportunistic infection. Clinical examination revealed a subcutaneous, 2 × 2-cm, firm, violaceous nodule with normal surrounding skin (Figure 1). He had no other similar lesions. There was no regional lymphadenopathy. The rest of the physical examination was normal. His hemogram was normal, as were his computed tomographic chest and abdominal ultrasound scans. He underwent fine-needle aspiration of the nodule, which was reported to have inflammatory cells and a few fungal hyphae. He subsequently underwent excision of the nodule, which was sent for microbiological evaluation. The finding was reported to be *Fusarium solani*.

**Figure 2:**
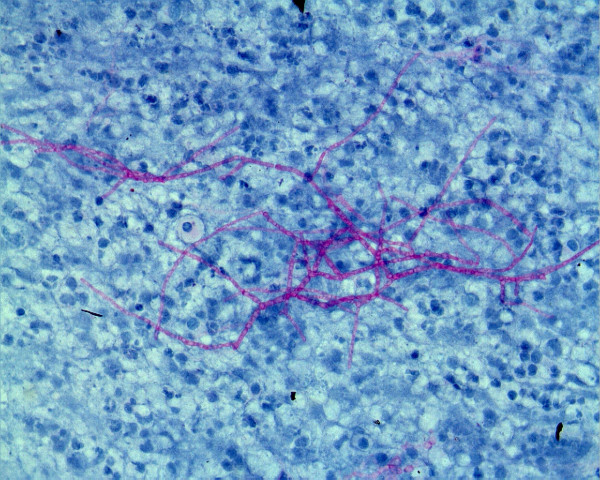
**Periodic acid-Schiff stain-positive spores of *Fusarium solani***.

A biopsy sample was cultured for fungi on Sabouraud dextrose agar without cycloheximide and was incubated at 25°C in air for four days. It grew whitish-gray cottony colonies suggestive of *Fusarium *spp. Successive subcultures performed on potato dextrose agar in the dark stained with periodic acid-Schiff showed sickle-shaped, multiseptated macroconidia, and one- to two-celled microconidia had formed from unbranched phialides, conidiophores and chlamydospores typical of *Fusarium solani *(Figure 2).

**Figure 3:**
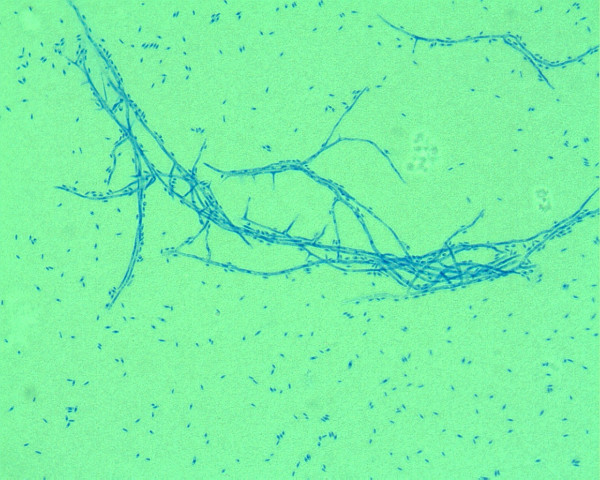
***Fusarium solani *identified by immunohistochemical staining**.

Subsequently, species identification was further performed using immunohistochemistry (Figure 3). The patient was successfully treated with surgical excision of the lesion followed by four weeks of oral voriconazole treatment.

## Discussion

*Fusarium *species are ubiquitous and may be found in the soil and air and on plants. In humans, *Fusarium *species cause disease that is localized, focally invasive or disseminated. The pathogen generally affects immunocompromised individuals, with infection of immunocompetent persons being rarely reported. Localized infection includes septic arthritis, endophthalmitis, osteomyelitis, cystitis and brain abscess. In these situations, a relatively good response may be expected following appropriate surgery and oral antifungal therapy. Disseminated infection occurs when two or more noncontiguous sites are involved [5]. The skin can be an important and early clue to diagnosis, since cutaneous lesions may be observed at an early stage of the disease. Typical skin lesions may be painful red or violaceous nodules, the center of which often becomes ulcerated and covered by a black eschar. The multiple necrotizing lesions are often observed on the trunk and the extremities [6].

Our patient had a single, localized nodule that was treated successfully with surgical excision and antifungal therapy. He did not have any signs of disseminated infection. At the last follow-up appointment, he had no symptoms of any disseminated fungemia. Amphotericin has been the drug of choice to treat most fungal infections. The use of azoles, namely, voriconazole, posaconazole and ravuconazole, has also been found to be promising [7]. As the patient was a renal transplant recipient, we chose to use voriconazole to treat him as it has shown good response in most zygomycoses.

## Conclusion

Opportunistic infections in transplant recipients can be life-threatening. *Fusarium *infections are recognized more often, and unless they are diagnosed and treated early, they can be a cause of significant morbidity and mortality.

## Consent

Written, informed consent was obtained from the patient for publication of this case report and accompanying images. A copy of the written consent is available for review by the Editor-in-Chief of this journal.

## Competing interests

The author declares that they have no competing interests.

## Authors' contributions

JSB analyzed and interpreted the patient's data and was involved in writing the manuscript. CSJ was involved in drafting the manuscript.
